# Using Video Telehealth to Support Family Caregivers of People With Dementia

**DOI:** 10.1093/geroni/igaa057.2282

**Published:** 2020-12-16

**Authors:** Joleen Sussman, Lauren Moo, Michele Karel

## Abstract

In 2030, predictions indicate that dementia will affect 75 million people worldwide and increase to 132 million by 2050. Persons’ with dementia (PWD) associated behavioral changes are highly correlated with caregiver burden. Caregivers of PWD commonly report concerns regarding personal and home safety, meaningful activities, advance care planning, and evaluation and diagnosis of dementia of the PWD. Further, caregivers’ emotional response to PWD challenging behavior has greater influence than the actual behavior on decisions to place PWD in a nursing home. Caregiver intervention reduces behavioral and psychological symptoms in the PWD, the caregiver’s emotional distress from these symptoms, and cost to healthcare systems. Yet, one in four dementia caregivers are not receiving dementia support services. Difficulty attending in person clinic-based appointments may be one barrier to caregivers engaging in treatment. This symposium highlights telehealth approaches, by various disciplines (Geriatrician, Neurologist, Geriatric Psychiatric, Geropsychologist, and Occupational Therapist), across urban and rural settings to address caregiver needs and improved access to care. The first presentation will focus on education of rural caregivers of PWD and increased connection to services (Sussman et al). The second presentation will focus on Video to home dementia visits for caregivers (Gately & Moo). The third study will focus on rural tele dementia caregiver support groups and effects on caregiver burden (Rossi et al). The final study will describe co-occurring caregiver and PWD telehealth groups (Thielke & Fredrickson).

